# Reducing stigma and improving access to care for people with mental health conditions in the community: protocol for a multi-site feasibility intervention study (Indigo-Local)

**DOI:** 10.1186/s13033-024-00649-3

**Published:** 2024-11-18

**Authors:** Maya Semrau, Petra C. Gronholm, Julian Eaton, Pallab K. Maulik, Bethel Ayele, Ioannis Bakolis, Gurucharan Bhaskar Mendon, Kalpana Bhattarai, Elaine Brohan, Anish V. Cherian, Mercian Daniel, Eshetu Girma, Dristy Gurung, Ariam Hailemariam, Charlotte Hanlon, Andy Healey, Sudha Kallakuri, Jie Li, Santosh Loganathan, Ning Ma, Yurong Ma, Amani Metsahel, Uta Ouali, Nahel Yaziji, Yosra Zgueb, Wufang Zhang, Xiaotong Zhang, Graham Thornicroft, Nicole Votruba

**Affiliations:** 1 Centre for Global Health Research, Brighton and Sussex Medical School, Brighton, UK; 2 Centre for Global Mental Health, Health Service and Population Research Department, Institute of Psychiatry, Psychology and Neuroscience, King’s College London, London, UK; 3 Centre for Implementation Science, Health Service and Population Research Department, Institute of Psychiatry, Psychology and Neuroscience, King’s College London, London, UK; 4 Centre for Global Mental Health, London School of Hygiene and Tropical Medicine, London, UK; 5 CBM Global, Cambridge, UK; 6 George Institute for Global Health, New Delhi, India; 7 University of New South Wales, Sydney, NSW Australia; 8 Manipal Academy of Higher Education, Manipal, India; 9 Department of Preventive Medicine, School of Public Health, Addis Ababa University, Addis Ababa, Ethiopia; 10 Department of Biostatistics and Health Informatics, Institute of Psychiatry, Psychology and Neuroscience, King’s College London, London, UK; 11 Department of Psychiatric Social Work, National Institute of Mental Health and Neurosciences, Bengaluru, India; 12 Transcultural Psychosocial Organization (TPO) Nepal, Pokhara, Nepal; 13 Department of Psychiatry, School of Medicine, College of Health Sciences, Addis Ababa University, Addis Ababa, Ethiopia; 14 Health Service and Population Research Department, Institute of Psychiatry, Psychology and Neuroscience, King’s College London, London, UK; 15 The Affiliated Brain Hospital of Guangzhou Medical University, Guangzhou, China; 16 National Institute of Mental Health and Neurosciences, Bengaluru, India; 17 Peking University Sixth Hospital, Peking University Institute of Mental Health, Beijing, China; 18 Department of Psychiatry A, Razi University Hospital, Manouba, Tunisia; 19 Research Laboratory LR18SP03, Tunis, Tunisia; 20 Faculty of Medicine of Tunis, University of Tunis El Manar, Tunis, Tunisia; 21 Nuffield Department of Women’s & Reproductive Health, University of Oxford, Oxford, UK; 22 The George Institute for Global Health UK, London, UK

**Keywords:** Mental health, Stigma, Access to care, Community awareness, Low- and middle-income countries, Protocol

## Abstract

**Background:**

Stigma and discrimination towards people with mental health conditions by their communities are common worldwide. This can result in a range of negative outcomes for affected persons, including poor access to health care. However, evidence is still patchy from low- and middle-income countries (LMICs) on affordable, community-based interventions to reduce mental health-related stigma and to improve access to mental health care.

**Methods:**

This study aims to conduct a feasibility (proof-of-principle) pilot study that involves developing, implementing and evaluating a community-based, multi-component, awareness-raising intervention (titled Indigo-Local), designed to reduce stigma and discrimination and to increase referrals of people with mental health conditions for assessment and treatment. It is being piloted in seven sites in five LMICs—China, Ethiopia, India, Nepal and Tunisia—and includes several key components: a stakeholder group workshop; a stepped training programme (using a ‘Training of Trainers’ approach) of community health workers (or similar cadres of workers) and service users that includes repeated supervision and booster sessions; community engagement activities; and a media campaign. Social contact and service user involvement are instrumental to all components. The intervention is being evaluated through a mixed-methods pre-post study design that involves quantitative assessment of stigma outcomes measuring knowledge, attitudes and (discriminatory) behaviour; quantitative evaluation of mental health service utilization rates (optional, where feasible in sites); qualitative exploration of the potential effectiveness and impact of the Indigo-Local intervention; a process evaluation; implementation evaluation; and an evaluation of implementation costs.

**Discussion:**

The output of this study will be a contextually adapted, evidence-based intervention to reduce mental health-related stigma in local communities in five LMICs to achieve improved access to healthcare. We will have replicable models of how to involve people with lived experience as an integral part of the intervention and will produce knowledge of how intervention content and implementation strategies vary across settings. The intervention and its delivery will be refined to be feasible and ready for larger-scale implementation and evaluation. This study thereby has the potential to make an important contribution to the evidence base on what works to reduce mental health-related stigma and discrimination and improve access to health care.

## Background

People with mental health conditions are often stigmatised and discriminated against in their local communities across the globe [1], including those with common mental disorders such as depression or anxiety, and those with severe conditions like schizophrenia, and substance use disorder. Stigma was defined by Goffman, one of the leading scholars of the last century on stigma, as a “deeply discrediting” attribution that reduces a person “from a whole and usual person to a tainted, discounted one” [2]. Since then, various types of stigma have been identified. Public stigma, for example, involves the stereotypes, negative attitudes and discriminatory behaviour among community members, or even health staff or family members, who may stigmatise a person due to some characteristic. This concept can be broken down into problems of knowledge (misinformation), problems of attitudes (prejudice) and problems of behaviour (discrimination) [3, 4]. Discrimination (or experienced stigma) is the behavioural expressione of stigma, i.e. the subjective experience of discrimination, exclusion or devaluation faced by a person due to a particular attribute. Self-stigma or internalized stigma becomes established when affected people accept the discrediting beliefs and prejudices held against them and lose self-esteem, leading to feelings of stress, shame, hopelessness and depression, a sense of alienation and social withdrawal [5].

Stigma (and discrimination) has far-reaching consequences for people with mental health conditions and has even been described by affected people as worse than the mental illness itself [4, 6]. It can have a range of negative impacts in terms of distress/stress, social exclusion and wellbeing, reduced employment opportunities and poverty, relationship difficulties [7], as well as poor access to health care and reduced healthcare-seeking behaviours [8–11]. These impacts can be direct or indirect, the latter mediated by various factors, such as self-stigma (for example, not applying for a job due to expectations of failure following internalisation of stigma) or lack of social support [4]. Whilst the actual experience of stigma itself seems to be similar across settings, stigma processes are complex and may be culturally influenced in terms of ‘what matters most’ in a particular context, in regard to the cultural concepts of conditions, cultural perceptions of their causes, and cultural determination of values [4, 12], highlighting how important it is for stigma reduction interventions to be adapted to the culture within which they are being implemented [13].

Over recent years there has been an increasing number of small-scale and short-term stigma reduction interventions published [14–18], with several systematic reviews examining their effectiveness [19–28]. Overall, these reviews have demonstrated that there are a number of education-based (addressing myths and misconceptions) and social contact-based (involving direct or indirect interactions with people with the stigmatised condition) interventions that produce small to moderate effects on stigma reduction in the short- to medium-term. Only a small percentage of these have been published from low- and middle-income countries (LMICs) [26], though one of the newer systematic reviews on the topic [19] found that effective mental health stigma reduction interventions in LMICs had increased in quantity and quality over recent years. The same review reported that research was limited to a small number of LMICs, that there was a lack of robust research designs, as well as a high number of short-term interventions and follow-up, and nominal use of local expertise in developing interventions or the cultural adaptation of interventions. Furthermore, the authors found minimal mention of social contact interventions despite existing strong evidence for them, concluding that more research and further translation/application of research findings are needed to address these issues [19].

There has also been a paucity of research published in LMICs evaluating the effectiveness of interventions aimed at reducing stigma and discrimination in the local community [19, 24, 29–31]. Even though community awareness-raising is commonly included in programmes working with marginalised or stigmatised groups, there is a significant lack of evidence about whether awareness-raising strategies alone are effective in reducing stigma in the community, particularly in regard to changes beyond knowledge, covering the essential areas of attitudes and behaviour. Changing attitudes and behaviour is recognised to be a complex process, and interventions focusing on increasing knowledge through education or teaching alone are not likely to be effective in changing behaviours. There is evidence that social contact interventions are one of the most effective ways in which to facilitate behaviour change, such as reduced discriminatory actions by community members or increased help-seeking behaviours by affected persons [3, 19]. Active interaction with a person who has lived experience of mental health issues appears to be more effective than passive interaction, though the delivery method (e.g. in person vs. virtual) seems to make less of a difference [4].

Previous work has shown increased mental health service utilisation following an awareness-raising programme in a low-resource setting in South-East Nigeria [32]. Between 2011 and 2013 Amaudo Itumbauzo, a civil society organisation working in mental health in South-East Nigeria, developed and implemented a mental health awareness-raising intervention [32]. The programme attempted to change community knowledge and attitudes towards people with mental health conditions and increase utilisation of their Community Mental Health Programme, which works within three States in South-East Nigeria to integrate mental health into health services at the local government level. The intervention involved training volunteer Village Health Workers to engage with key community gatekeepers (traditional leaders, churches, women’s and youth groups) and share messages about mental health that challenged common misconceptions, and also involved a media and radio information campaign. This was a refined version of an earlier programme [33] and was implemented in partnership with CBM (an international non-governmental organisation (NGO)). The programme was shown to significantly increase attendance at primary care clinics under the Amaudo Community Mental Health Programme.

The Indigo-Local study described here builds on the Amaudo programme and extends it to other settings. The study is part of the larger Indigo Partnership programme, which involves developing and piloting a range of mental-health-related culturally-adapted, multi-level stigma reduction interventions across a variety of target populations in seven sites across five LMICs in Africa and Asia [34]. The Indigo Partnership arose out of the Indigo Network, which is an international network of researchers committed to the promotion of mental health by reducing stigma and discrimination related to mental illness [35]. Since the previous Amaudo programme [32] did not include a specific role for social contact interventions with people living with mental health conditions, the Indigo-Local study developed an intervention that added this component to awareness-raising through media and information-sharing by professionals. The Indigo-Local intervention therefore contains the elements previously used in the Amaudo programme [32], but deliberately adds an element of social-contact service user testimony, because of the clear evidence that has emerged since then of the impact of personal testimony and the direct involvement of people with lived experience of mental health conditions in changing attitudes to mental health conditions, in reducing stigma towards health-seeking, as well as reducing social distance and experience of discrimination [3, 4]. Furthermore, the Indigo-Local intervention incorporates an awareness-raising media campaign that follows recent understanding of effective means of sharing information in the community [17, 18, 36, 37]. The Indigo-Local study thus focuses on the ability of the intervention to reduce stigma and discrimination, using broader stigma measures that capture knowledge, attitudes and behaviour. In addition, it includes service utilisation rates as a secondary outcome, based on evidence from the Amaudo programme [32] and on the assumption that the reduction in misinformation and resultant change in explanatory models for mental health conditions from traditional spiritual causation to health causes can be a driver for increased service uptake [38].

The aim of the Indigo-Local study is therefore to conduct a feasibility (proof-of-principle) pilot study that involves developing, implementing and evaluating a community-based, multi-component awareness-raising intervention designed to reduce stigma and discrimination and increase referrals of people with mental health conditions (which may include common and severe mental disorders, and substance use disorder, depending on what is appropriate within sites) for assessment and treatment in all seven of the Indigo Partnership sites.

## Methods

### Study design and objectives

The Indigo-Local feasibility pilot study aims to:

1. Develop a community-based awareness-raising intervention (Indigo-Local) that involves training community health workers (or similar cadres of workers) and mental health service users, alongside community engagement activities and a media campaign, designed to: (1) reduce public stigma amongst community health workers (in terms of knowledge, attitudes and discriminatory behaviour) and the wider community, and self-stigma amongst service users (in terms of knowledge, attitudes, experienced stigma/discrimination and stress due to stigma), and (2) increase referrals of people with mental health conditions for assessment and treatment.
2. Implement and pilot the Indigo-Local intervention in a small feasibility (proof-of-principle) platform activity using a pre-post mixed-methods study design in seven sites in five LMICs, to evaluate procedures for a subsequent fully-powered study comparing the clinical and cost-effectiveness of Indigo-Local in: (1) reducing stigma and discrimination amongst trained community health workers (or similar cadres of workers), the wider community and service users, and (2) increasing mental health service uptake.

**Fig. 1:**
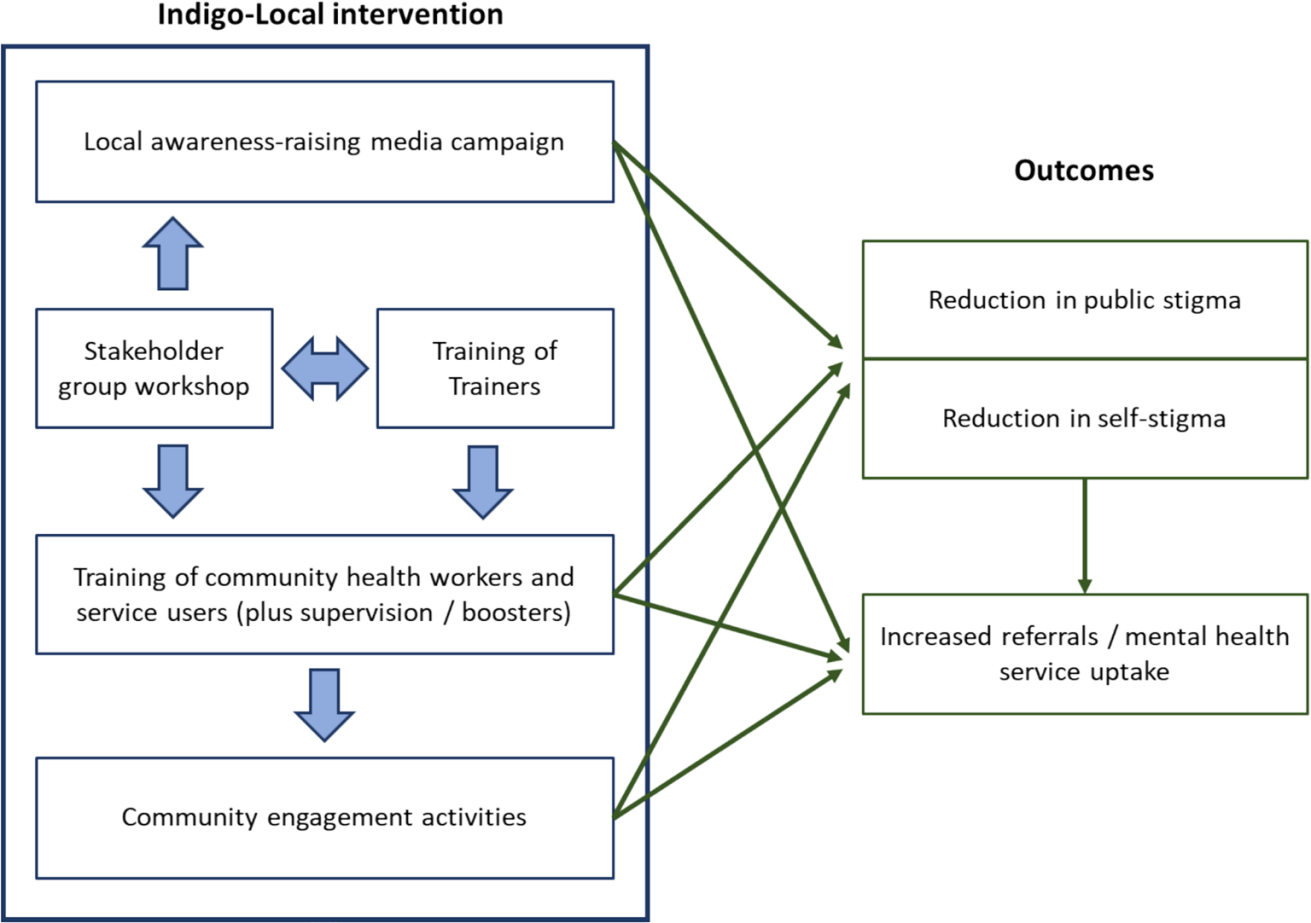
Overview of Indigo-Local intervention and its proposed pathways with outcomes

See Fig. 1 for a visual overview of the Indigo-Local intervention and its proposed outcomes. The Indigo-Local intervention includes all of the following elements: Training of Trainers (ToT), with the Indigo-Local research leads training site teams; stakeholder group workshop (with local stakeholders, e.g. health service leaders, members of service user organisations etc.) which includes development of messages for an anti-stigma campaign, designed to counter local stigmatising ideas and attitudes; training by site teams of community health workers (or similar cadres of workers) and service users; engagement activities in the community; supervision meetings/booster trainings; and a media campaign in the community. These will be described in more detail further below. Whenever the term ‘intervention’ is used in this paper, this refers to all of these components.

### Setting

The Indigo-Local feasibility pilot study is being carried out in seven sites in five LMICs [34, 35], i.e. two sites in China (Beijing and Guangzhou), two sites in India (Bengaluru and Delhi National Capital Region), Ethiopia, Nepal and Tunisia. See Table 1 for further details about the study setting/location for each of the seven sites. The study sites have been selected based on accessibility, appropriateness and feasibility, and where possible entail a distinct region or neighbourhood.

**Table 1:** Settings, participants and design of Indigo-Local intervention in the seven study sites

Study site	Location of training/intervention implementation	Trainers used in training (worker type and number of trainers)	Recipients of training (participant type and target sample size)^a^	Media campaign components
China, Beijing	Xicheng district, Tongzhou district, Chaoyang district (all urban)	3 site team members	16–18 community mental health workers who are responsible for follow-up (home) care and working at primary medical facilities; 3 service users	Posters and social media (WeChat) for at least 1 month
China, Guangzhou	Tianhe district (urban)	2 site team members	12 community mental health social workers; 4 service users	Leaflets and social media (WeChat) for at least 1 month
Ethiopia	Sodo and South Sodo districts, Gurage Zone, South-Central Ethiopia (rural)	3 site team members	15 community-based health extension workers; 2 service users	None
India, Bengaluru	Ramanagara district (rural)	3 site team members	86 Accredited Social Health Activists (ASHAs), 5 primary health care officers; 6 service users	Distribution of brochures and posters by ASHA workers for 1 month
India, Delhi National Capital Region	Atmadpur and Mewla Maharajpur primary health centre areas (urban)	3 site teams members	11 ASHAs; 5 service users	Screening of videos in the community and use of flipbooks by ASHAs during their routine visits, for 3 months
Nepal	Gandaki Province, Syangja District, Arjunchaupari Rural Municipality, Central Nepal (rural)	1 site team member and 1 psychosocial counsellor	20 Female Community Health Volunteers (FCHVs) from four primary health care centres; 2 service users	Flyers distributed by FCHVs and hoarding boards/billboards placed in strategic locations such as schools, marketplaces etc., run actively for 4 months
Tunisia	Testour Municipality, Governorate of Beja, Northern Tunisia (rural)	3 site team members and 2 residents in Psychiatry	18 well-regarded members of the community of Testour: social workers, religious leaders, members of local NGOs, members of the local radio, teachers; 1 service user	Flyers, posters in strategic locations, social media posts, radio broadcasts about mental health and stigma, for 3 months

^a^Note that the service users listed here were both recipients of the training and also provided the social contact element of the training, i.e. provided lived testimonies.

The Indigo-Local intervention is being implemented in community settings within the seven study sites, such as in public spaces or community facilities. The training elements of the intervention are being conducted either within health, community, private or work spaces as appropriate, depending on the local contexts. For ethical reasons, mental health services need to be in place in the settings in which the Indigo-Local intervention is being implemented, given the likely stimulation of and anticipated increase in help-seeking.

### Participants

A wide range of stakeholders will be involved in the Indigo-Local feasibility pilot study in each of the seven sites. This may include local key stakeholders such as health service leaders and/or members of service user organisations, community health workers or similar cadres of workers, (mental) health and/or site staff, service users and their caregivers. Table 2 shows an overview of the different study activities that each of the participant groups are involved in.

**Table 2:** Study activities according to participant groups within the Indigo-Local feasibility pilot study

Participants	Intervention implementation activities						Mixed-methods evaluation of intervention
	Training of Trainers (ToT)	Stakeholder group workshop	Training of community health workers (or similar cadres of workers) and service users	Community engagement activities	Supervision meetings/booster trainings	Media campaign	
Local stakeholders, e.g. health service leaders, members of service user organisations etc.		✔				✔	
Indigo-Local research leads	✔ (as trainers)						
Site teams	✔ (as trainees)		✔ (as trainers)		✔ (as supervisors/trainers)	✔	✔
Community health workers or similar cadres of workers			✔ (as trainees)	✔ (as facilitators)	✔ (as supervisees/trainees)	✔ (some sites)	✔
Mental health service users		✔	✔ (as trainees; service user contact element)	✔ (as facilitators)	✔ (as trainees)	✔ (service user contact element)	✔

#### Inclusion and exclusion criteria

All participants of the Indigo-Local feasibility pilot study will be at least 18 years of age and have to freely consent to participate. We will review mental capacity to consent where a concern is raised, but seek to respect preference of the service user in all cases. For all groups, sampling further aims to achieve adequate sample variability with regard to gender and age group of participants. Further details about participant eligibility are outlined below in the section on the key components of the Indigo-Local intervention. We are excluding anybody who is at risk of a psychiatric emergency, who may not be able to provide consent, or who may not be able to perform the intervention and research activities.

#### Recruitment

All participants of the study will be sampled purposively by each of the site teams. All participants will be identified and approached by either the implementing partners in the country sites, or by the local health service leaders or similar key stakeholders, to engage them to participate in the study. Where possible, contact regarding the study will be conducted by an impartial third-party individual (i.e. not the participants’ clinician [for service users] or staff managers [for health workers], but instead, for example, a recruitment officer, research assistant, PhD student, or clinic administrator, depending on site resources).

#### Sample sizes

Sample sizes will vary between the seven study sites, which will be determined within sites depending on feasibility and the local resources available, as well as the size of the site—see Table 1 for further details. We plan to recruit a minimum number of ten community health workers (or similar cadres of workers, depending on the local context) and service users in total for training in each of the seven sites. If possible, of the total number of participants recruited for training, a minimum of 15–20% should be service users in each of the sites (and the rest community health workers or similar cadres of workers). All trained community health workers and service users should ideally be involved in the quantitative evaluation of the Indigo-Local intervention, and a sub-set of them are also taking part in the qualitative evaluation per site (depending on site feasibility). In addition, two to three people will receive the ‘Training of Trainers’ training in each of the seven study sites, and between five and 20 participants will take part in the stakeholder group workshop per site (depending on local feasibility).

Since the Indigo-Local study is being conducted on a proof-of-principle feasibility basis, it is not appropriate or necessary for sample sizes for the quantitative evaluation elements to be guided by power calculations; rather will there be a minimum of 30–50 participants in total across the sites. This is in line with recommendations for sample sizes of between 24 and 50 for feasibility studies [39–42]. The intention is not to formally test for pre-post differences in the sample, but we will instead examine the effect size and direction of change, which could guide the sample size for a future full-scale study. Further evaluation data will be collected through qualitative means, using a data saturation approach as is usual in qualitative research, for which the sample sizes outlined are appropriate.

## Indigo-Local intervention

### Principles guiding the Indigo-Local intervention

See Box 1 for the principles guiding the Indigo-Local intervention as its ‘essential ingredients’, based on the Amaudo Mental Health Awareness Programme in South-East Nigeria [32] and other work since then [19, 43, 44].

**Box 1:** Principles guiding the Indigo-Local intervention

*Service user involvement and incorporating social contact elements* (e.g. sharing of ‘lived testimonies’), both within the training components and community engagement activities. Service user involvement is a key element of the Indigo-Local intervention. Service users are being trained together with community health workers (or similar cadres of workers) where possible. The intention is for direct social contact to be incorporated within all training elements.
*Gaining buy-in from health system leaders* and linking the awareness-raising to the existing services, for example by using existing personnel and mapping the training to specific health system infrastructures.
*Involving front-line community-based health workers*, such as community health workers or similar cadres of workers, who know their communities well, are trusted within the communities, and are familiar with community education and health mobilisation—in many low- and middle-income settings these will be volunteer workers, though not always. In addition to the principle that they should be well known by their communities, and are intimately familiar with community cultural perspectives, it is efficient to use existing cadres of personnel to implement health initiatives linked to novel services or scale-up efforts.
*Developing key messages and materials with key community members* who understand the local community beliefs and attitudes, as well as the local health service context. This is done by holding a stakeholder workshop with mental health experts, community health workers (or other delivery agents), community members and people with lived experience. The local underlying stereotypes and beliefs are documented, and counter-points to the misconceived or stigmatising ideas developed. They are then formulated in a way that will be understood by the local community.
*Achieving scale-up through a stepped process* of ‘Training of Trainers’, followed by training of community health workers (or similar cadres of workers). This also allows the building of relationships and key referral links for subsequent services (both through networking of participants and increasing awareness amongst participants of available resources). As an example, in the Amaudo study, the community psychiatric nurse carried out the training for the community health workers in their catchment area.
*Linking the training in each site with activities mobilising key leaders and decision-makers* in the community. In the Amaudo study, this was done through visits to community leaders ahead of the training to elicit their support for the project, and an opening ceremony on the first day of training.
*Using media as a means of reinforcing the community engagement activities in each area*. In the previous study, this comprised radio and TV reports, as well as ‘jingles’ on local radio informing communities of the existence of the clinics. These were timed to coincide with the community engagement. This was a means of rolling out the stigma reduction materials; it was not a mandated activity.
*Implementing/establishing a continuous support process* of providing ongoing basic supervision, continued linkage with trained community health workers and motivational strategies (e.g. biannual meetings, and awards for the most effective workers.)

### Key components of the Indigo-Local intervention

**Fig. 2:**
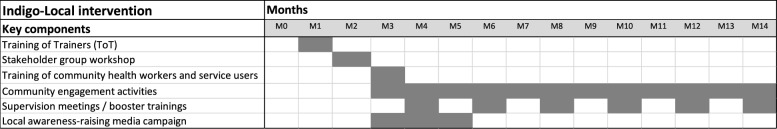
Example timeline and sequence for the key components of the Indigo-Local intervention

The key components of the Indigo-Local intervention are outlined below in more detail. Each of these key components will be carried out in each of the seven study sites. Figure 2 shows an example timeline and sequence for the key components of the intervention.

#### Training of Trainers

The plan for future Indigo-Local interventions is for the ‘Training of Trainers’ (ToT) to be conducted for 5 days residential, whereby master trainer(s) are trained to train people to conduct the community health worker/service user training. This ToT training should include a direct (e.g. a service user provides a ‘lived testimony’ in person) or indirect (e.g. showing a video of a person talking about their experiences) contact element with service user(s). However, since this is a feasibility study with small sample sizes and since the teams in each site are mental health stigma experts with prior knowledge on the topic, in this study an online ToT programme is being carried out, in which the Indigo-Local research leads train site teams to conduct the community health worker/service user training in around 1 day through a series of online training videos and seminars.

A minimum of two to three people should take part as recipients of the ToT training in each site. Ideally, these participants would be expected to have some mental health knowledge. Recipients of the ToT are taught to train the community health workers (or similar cadres of workers) and service users about mental health and stigma, and how to share mental health related messages in community forums (e.g. community meetings), for example to give advice about the location and availability of mental health services (including opening times), referral methods, follow-up and monitoring of service users in the community, and the costs involved. The training also includes a brief overview and materials to understand effective implementation strategies for the community engagement activities and media campaign.

#### Stakeholder group workshop

A stakeholder group workshop is being conducted for the duration of half up to 1 full day in each of the study sites. In each site between five and 20 participants are joining, including relevant local stakeholders, such as health service leaders, members of service user organizations, local community groups or NGOs, community workers, health staff, service users, traditional healers, religious leaders etc. Local health service leaders are purposively selected and invited into the study by the local research teams based on the following characteristics: they should hold a leadership role at their institution within health services in the site, ideally within mental health services (or have a good working knowledge of mental health issues). Any other local stakeholders should be people or groups who advocate and manifest the interest and will of mental health service users in the community, or who are engaging or supporting people seeking mental health care.

The aims of the stakeholder group workshop are to: (a) bring all key stakeholder groups together to establish the project team, build relationships, and ensure buy-in from the beginning; (b) advise on the local context, training needs and the local media landscape; (c) review, refine and adapt the training materials and translate them into the local language (where needed/appropriate)—for consistency and fidelity, the material templates have been developed centrally (based on the materials used in the Nigeria study, provided by Amaudo [32]), which allows for sharing of evidence-based practice; however, these materials are being adapted by each of the sites to cover local cultural beliefs and specific issues related to the area of intervention; (d) plan and define the media strategy and clarify its messages; (e) help in planning the training, including identifying which cadres of workers to train—it is crucial that this is done carefully to maximise the efficacy and retention of those trained, and involves defining in advance what is expected, post-training, of the trainees (e.g. to hold community forums, to identify and refer patients in their community etc.); and (f) help in planning the implementation of the intervention, including refining details of the intervention to match local services, resources and needs, and deciding on the most appropriate way(s) to raise awareness in the community through the community engagement activities. The stakeholder group workshop builds on detailed formative work already completed previously in study sites as part of the Indigo Partnership [34, 35].

#### Training of community health workers and service users

Community health workers (or similar cadres of workers) and service users are being trained over a minimum of 2 days (for resource-limited settings) up to an ideal maximum of 5 days in each of the sites. Training could be conducted over successive days or in separate blocks over a few weeks, depending on feasibility and the local context within sites. At least ten participants in total per site will be trained, within or near their local communities.

Community health workers or similar cadres of workers who are trained are selected based on the following characteristics:

- they should be well-respected members of the local community;
- should know their communities well and be intimately familiar with community cultural perspectives;
- should be familiar with community education and mobilisation;
- should be part of existing cadres of personnel if possible, for instance Accredited Social Health Activists (ASHAs), female community health volunteers (FCHVs), government officers, faith-based group leaders etc.

Careful choice of such workers was found to be crucial for good results, coordination and sustainability during the previous Amaudo programme in Nigeria [32].

Eligible mental health service users to be trained alongside community health workers (or similar cadres of workers) can include any person seeking care from and using a mental health service. We expect to involve people with a range of diagnoses from common mental illness (depression, anxiety) to more severe mental illness (bipolar disorder, psychosis) or harmful substance use. These service users who are being included as recipients of the training should be able, willing and feel safe to discuss their own experience of living with a mental health condition as well as their own mental health service use. Ideally this should be somebody from the local community, though service users from elsewhere can be involved if necessary (recognising that for some, speaking in their own community may pose greater challenges or risks). In sites where this is deemed to be appropriate and beneficial service users’ caregivers may also be involved in the training.

The training is facilitated by the recipients of the ToT within or near their local communities, who train the community health workers (or similar cadres of workers) and service users (and possibly their caregivers where appropriate). Community health workers and service users should ideally be trained together to reinforce the social contact element of the training (in that case, both groups will likely need to be briefed before and debriefed after the training), but if this is considered not to be possible or good practice in sites (e.g. because of power dynamics, social hierarchies etc.), the two groups could also be trained separately. If such direct in-person contact is not possible during the training, then the social contact element could also be done through indirect contact, for example video or online materials that could have been developed previously (e.g. Time to Change Global [43–45] or other locally relevant materials).

The training content includes mental health and stigma, awareness-raising, i.e. how to spread messages of mental health (services) in the selected community, and how to conduct outreach and referrals (for which the pathways will be contextualised by sites). The training takes more of an approach focused on human rights and recovery, rather than primarily presenting mental health as a ‘brain problem’. See Table 3 for further details on the training content. Sites are required to culturally adapt the training and complement it with contextually relevant information from other sources, using both the Ecological Validity Model (EVM) [46] and the ‘Template for intervention description and replication’ (TIDieR) checklist [47] as frameworks.

**Table 3:** Content for Indigo-Local community health worker/service user training

Module title	Aims and objectives	Teaching methods
1. Mental health and mental illness	Aim: Learn about mental health and mental illness Objectives Recognise behaviour that is a cause for concern Recognise the features of some mental illnesses Understand that people with mental illness need help from health care professionals	Introductory lecture Interactive activities What is normal behaviour; what is a cause for concern? Small group discussion—Learning about mental health problems Carousel—How can we teach our communities about mental health and mental illness? Quiz—Mental health and mental illness
2. Human rights and mental illness	Aim: Learn about human rights and mental illness Objectives Understand five human rights laws and how these relate to people with mental illness Feel equipped to help prevent human rights abuses in the community	Introductory lecture Interactive activities What would you do if…? How do human rights laws relate to people with mental illness? Case study discussion Prisons and mental illness quiz
3. Caring for people with mental illness	Aim: Learn about caring for people with mental illness in the community Objectives Understand the principles of mental health promotion and education Know how to refer somebody to a clinic Understand the principles of medication used to treat mental illnesses and recognise types of side-effects Understand the principles of monitoring recovery and assisting rehabilitation in the community	Introductory lecture Interactive activities Small group discussion—How can community health workers help? Referral to a health clinic Role play—Supporting recovery Quiz—What community health workers need to know about treatment
4. Stigma and mental illness	Aim: Learn about stigma and mental illness Objectives Understand the core problems in stigma Recognise examples of stigma Be aware of different ways to reduce stigma	Introductory lecture Interactive activities: Guest speaker—Living with mental illness Case study discussion Designing posters Group discussion—What does stigma and discrimination mean to you?
5. Practical steps	Aim: Learn about practical steps for promoting rights and reducing stigma Objectives Have a workable plan about how to teach their community about mental illness, promote rights and reduce stigma	Introductory lecture Interactive activities: Post-it notes—What I have learnt? Practical steps—Planning of stigma reduction activities in the community

#### Community engagement activities

The trained community health workers (or similar cadres of workers) and service users will then conduct community engagement activities (i.e. locally contextualised awareness-raising activities/engagement) in the local community within each of the sites, to address public stigma. This may be embedded within their usual role. The exact awareness-raising activities are intentionally left flexible for the sites to implement based on the local context, but the activities should include two-way engagement of the community in some way. For example, this may include community contact activities, speaking to community groups (e.g. faith locations, women’s groups, youth groups etc.), or at events or locations such as markets. Two-way engagement with community members is key, to distinguish it from the media campaign which is one-directional in that messages are conveyed to the community without any necessary direct response or interaction.

#### Supervision meetings/booster trainings

Supervision meetings for the trained community health workers (or similar cadres of workers) and service users will take place every two to 3 months, with brief booster trainings after three to 6 months and 6–12 months (if feasible in sites). Process data, for example on their level of activity in regard to mental health awareness-raising, could be collected as part of these sessions. Ideally these supervision meetings and booster trainings will be conducted by the same people who conducted the initial training.

#### Local awareness-raising media campaign

A media campaign targeted towards members of the community is being conducted over a minimum of a 1-month period (ideally longer), which starts at the same time as the training of the community health workers and service users. The format and messages of the media campaign depends on what is feasible and appropriate within each of the sites, but may include posters, flyers, newspaper articles, social media (WhatsApp, Facebook, Instagram, Twitter etc.), announcements or jingles in local radio or television etc. At least two different media outlets should be used in each site—see Table 1 for further details on this for each of the study sites.

The media campaign is being developed by the local site teams according to the local context. The content of the campaign is framed and phrased as such that it will aim to help increase public knowledge and improve attitudes and awareness around mental health conditions, and inform the community about the availability of mental health services, based on previous evidence about the nature and content of messaging for attitude change [17, 18]. The messages are linked to services and to the content of the training activities (e.g. myth-busting, information about available services etc.). The campaign messages should include an (indirect) social contact/‘lived experience’ element, such as a video or an interview with persons with lived experience (for which there are good examples available [43, 48]).

## Evaluation of Indigo-Local intervention

The evaluation of the Indigo-Local intervention will be conducted as a feasibility (proof-of-platform) pilot study using a mixed-methods design. This will involve quantitative evaluation of stigma outcomes; quantitative evaluation of mental health service utilization rates (optional, where feasible in sites); a qualitative evaluation exploring effectiveness of the intervention in terms of stigma outcomes and mental health service use, and an evaluation of the training; a process evaluation; an implementation evaluation; and an evaluation of implementation costs. These aspects are each described further below. An overview of these evaluations along with the time points for their assessment are provided in Table 4 (adapted from the SPIRIT flowchart; a populated SPIRIT checklist is provided as additional file [49–51]).

**Table 4:**
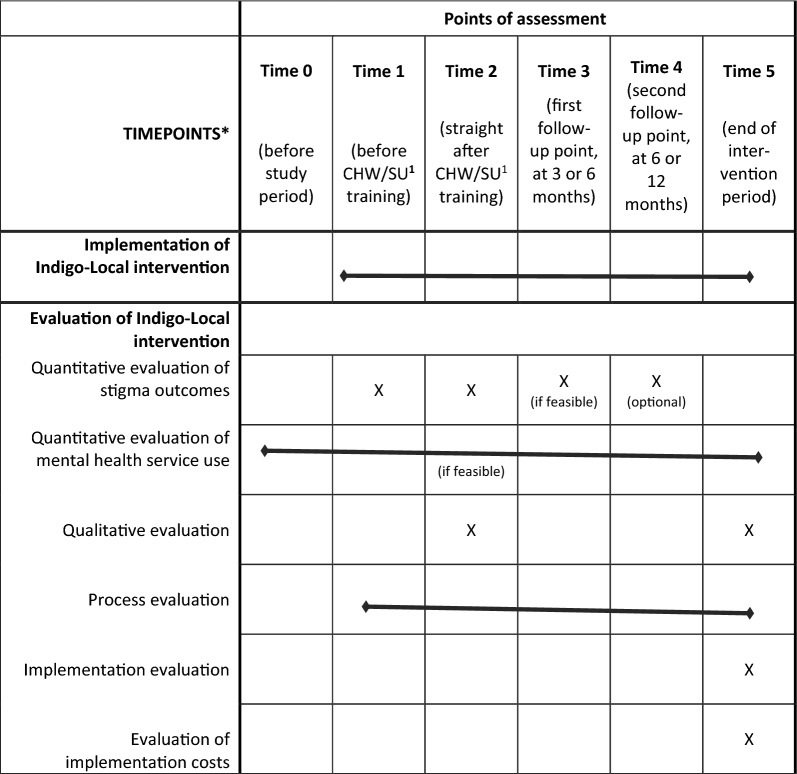
Overview of assessment time points for evaluation of Indigo-Local intervention

Note that where bars are indicated, this means that there is no defined time point for the particular implementation/evaluation, but rather is the implementation/evaluation carried out across the life time of the project.

^1^CHW/SU: community health workers/service users

The purpose of this mixed-methods approach is triangulation and complementarity between the quantitative and qualitative evaluations, which will run independently and concurrently overall, though at distinct time points (see Table 4) [52]. Both the quantitative and qualitative aspects equally form a core component of the study, i.e. are of equal status. Findings will be integrated at the point of the analysis of results [52].

### Quantitative evaluation of stigma outcomes

This involves pre vs. post assessment of quantitative scales to measure stigma and discrimination (in terms of knowledge, attitudes and (intended/expected) discriminatory behaviour) amongst the community health workers (or similar cadres of workers) and service users who receive the training, using the following quantitative questionnaires:

- Changes in knowledge about mental health conditions: The ‘Mental Health Knowledge Schedule’ (MAKS) [53] will be completed by the trained community health workers and service users. The MAKS has 12 items, which are each scored on a five-point Likert scale, with higher scores indicating higher levels of knowledge.
- Changes in (intended/experienced) discriminatory behaviour:
  - The ‘Reported and Intended Behaviour Scale’ (RIBS) [54] will be used to assess changes in intended behaviour by the trained community health workers. The RIBS contains eight items across two sub-scales, which are rated either as ‘yes/no’ response or on a Likert scale, with higher total score indicating higher willingness to interact with a person with lived experience of a mental health condition.
  - The shortened version of the ‘Discrimination and Stigma Scale’ (DISCUS) [55] will be used to assess changes in mental health service users’ experience of stigma and discrimination. The DISCUS has 11 items, which are rated on a four-point Likert scale, with higher scores indicating higher levels of discrimination. Service users who take part in the Indigo-Local training component will complete the DISCUS.
- Stress: The shortened 2-item version of the Stigma Stress Scale [56], for completion by the service users who take part in the training. Higher scores indicate higher levels of stress due to stigma, with total scores ranging between − 6 and 6.
- Changes in attitudes towards people with mental health conditions: Social Distance Scale (SDS) [57, 58], for completion by the trained community health workers (or similar cadres of workers) and service users. The SDS has 12 items, which are each rated on a six-item Likert scale, with higher scores indicating greater social distance. This scale is optional rather than obligatory for sites.

All scales have been validated and used in earlier studies across a wide range of countries worldwide [53–59]. They have already been adapted and translated by the site teams locally as part of the formative work within the Indigo Partnership [34, 35].

All scales will be completed at several time points (see Table 4). As a minimum, these data will be collected immediately before (Time 1) and after (Time 2) the community health worker/service user training. If feasible in sites, at least one further follow-up point will be included, ideally at 3 or 6 months (Time 3). Further follow-up assessment time points (e.g. at the time of the booster training sessions at 6 or 12 months) are optional depending on feasibility within sites (Time 4).

### Quantitative evaluation of mental health service utilization rates

Where feasible in sites (optional), this will be conducted to test the difference on mental health service utilization rates of the Indigo-Local intervention. In sites where this is feasible and appropriate, quantitative data that are routinely collected by health workers within the health system will be used to assess (at site-level) the following (or similar/related/proxy) outcomes:

- Total number of ‘new referrals’ to mental health services by the community health workers who participated in the training (e.g. by comparing to 1-year pre-intervention);
- Total uptake of mental health services, including total number of service users seen by mental health services (and % change), and new referrals to mental health services (and % change);
- Contact coverage (defined as service utilization taken from the programme records divided by the total population in need of services taken from prevalence surveys of the disorder), where feasible, i.e. where adequate data is available in the scientific literature for the site about the number of people who require mental health services (to act as denominator of contact coverage) [60].

If feasible, routine data should be collected (retrospectively) on a monthly basis for 1 year before the Indigo-Local intervention is implemented, and then on a monthly basis for a minimum of 1 year after the intervention is implemented (to assess the long-term impact of the intervention and also the impact of the supervision meetings/booster trainings).

Where feasible, data will be collected on previous referrals of patients, as well as on referral pathways/how referrals are made, for example referral by community mental health workers, self-referral following the media campaign etc.

### Qualitative evaluation

A qualitative evaluation will be conducted to assess the process and experience of implementation of the intervention components including training, to complement the quantitative findings. In-depth qualitative data will be obtained from community health workers and service users on the potential effectiveness of the Indigo-Local intervention in terms of stigma (knowledge, attitudes, behaviour) reduction, mental health service utilization rates (including referral rates), and the impact of the intervention amongst participants who received the training to deliver the intervention. The following will be explored qualitatively: (1) ways to improve the training; (2) changes in stigma, including possible explanations for changes in the quantitative outcomes/lack thereof, based on the directions of change observed; (3) information around possible changes in mental health service utilization rates; (4) other outcomes not covered by the quantitative measures, including any possible negative, unintended consequences. This will be done through focus groups and/or semi-structured interviews, ideally immediately after the training (i.e. Time 2) and/or at the end of the intervention period (i.e. Time 5); the data collection approach will be selected based on feasibility and appropriateness in each study site.

### Process evaluation

In addition, a process evaluation will be conducted at site-level, to record the exact implementation details of the Indigo-Local intervention in each of the sites. For this, process indicators will be collected using a specially-developed Excel file, employing the TIDieR checklist as framework [47]. This framework consists of 12 items, relating to the following aspects of an intervention: brief name, why (rationale), what materials (e.g. training materials), what procedure (e.g. types of activities), who provided (e.g. the training), how (e.g. in person or not), where (e.g. health facility, community), when and how much (e.g. how many times and when the community health workers/service users are involved in awareness-raising activities), tailoring, modifications, how well (planned), how well (actual).

### Implementation evaluation

Implementation of the Indigo-Local intervention will be evaluated at site-level with members of the seven site research teams. Semi-structured interviews will be carried out by the Indigo Partnership project coordination team with the research teams in each of the implementing sites at a minimum of one time point post-intervention. These interviews will collect information on the site teams’ implementation experiences and perceptions of the facilitators and barriers to implementation. Feasibility of the intervention will also be explored qualitatively via focused questions about this to the site teams. These interviews will be framed around an established implementation strategy framework, the updated Consolidated Framework for Implementation Research (CFIR) [61, 62].

Data for this will be analysed descriptively. Patterns in these data will be explored across and within sites, based on data of what types of implementation strategies were used, and how many strategies were reported to be used. Data on implementation facilitators/barriers will be synthesised narratively, guided by content analysis and thematic analysis principles. The results of this will be published separately.

### Evaluation of implementation costs

A cost analysis will be undertaken that will estimate the quantity of resource inputs and costs associated with intervention implementation activities across the seven study sites, in order to produce a cost estimate for the Indigo-Local intervention in the different sites. This will draw on data supplied by local site leads who will complete a costing pro-forma designed specifically for the Indigo pilot evaluation. This asks for quantitative information on staff time inputs, local pay rates and financial expenditures recorded against key implementation activities. The design of the pro-forma has been informed by an activity-based costing approach to assessing the cost implications of implementing health programmes, as outlined by Cidav et al. [63].

Estimates of total implementation costs and costs related to broad categories of implementation activity will be presented by study site. Costs will be presented in both local currency values and in US dollar purchasing power parity (PPP) adjusted values using appropriate PPP conversion factors published by the World Bank [64].

### Data management

REDCap [65] will be used for entry of quantitative data, with response fields for all items (including respondents’ socio-demographic characteristics, site characteristics and outcome variables). In each site a member of the local research team is identified, who is responsible for local data collection and data entry. The coordinating team at King’s College London will then export data from REDCap for data checking and cleaning. Data will be processed in accordance with the General Data Protection Regulation 2016 (GDPR). All data collected will be kept securely by the research team at King’s College London, in locked cabinets and offices as well as password-protected electronic files. Data will be shared between members of the research team using a secure file transfer service for transcription, translation and analysis of the data. We will keep unidentifiable data collected as part of this study indefinitely.

### Data analyses

The suitability of the measures will be examined, for instance for their distribution, and ceiling and floor effects. This is in line with the aims of this being a feasibility (proof-of-principle) pilot study.

For the quantitative data analyses, descriptive summaries such as total scores and simple counts will be performed, which will then be compared at the different time points, as well as the % change before and after the intervention is implemented (using chi-square tests). Primary and secondary outcomes will be analysed using mixed effects linear or logistic or Poisson regression models depending on the data type accounting for clustering due to repeated observations at three time points (Times 1, 2 and 3) in each site. Regression results will be pooled across countries using random effects meta-analysis, with a test for heterogeneity of regression coefficients being summarised using the I2 statistic [66]. All data analyses will be conducted with the use of STATA 17.

For the qualitative analyses, focused framework analysis, deducted based on the themes included in a specially-developed topic guide, will be carried out, with some inductive thematic analysis principles also applied with further bottom-up codes generated by sites where applicable and site teams identifying select key illustrative quotes to enrich the narrative analysis. The focus groups and/or semi-structured interviews will be audio-recorded and transcribed verbatim before being translated into English (where appropriate) and then analysed.

## Conclusions

Indigo-Local is a multi-site feasibility (proof-of-platform) pilot study, aiming to develop, implement and evaluate a community-based awareness-raising intervention designed to reduce mental-health-related stigma and improve access to mental health services in seven sites in five LMICs in Africa and Asia. The intervention includes several key components: a stakeholder group workshop; a stepped training programme (using a ToT approach) of community health workers (or similar cadres of workers) and service users that includes repeated supervision and booster sessions; engagement activities in the community; and a media campaign. The output of this study will therefore be a contextually adapted, evidence-based intervention to reduce mental health-related stigma in local communities to achieve improved access to mental health care. We will have replicable models of how to involve people with lived experience as an integral part of the intervention and will produce knowledge of how intervention content and implementation strategies vary across settings. The intervention and its delivery will have been refined to be feasible and ready for larger-scale implementation and evaluation. This study thereby has the potential to make an important contribution to the evidence base on what works to reduce mental-health-related stigma in local communities in LMICs.

## Data Availability

Not applicable.

## Abbreviations

ASHAs: Accredited Social Health Activists
DISCUS: Discrimination and Stigma Scale (short version)
FCHVs: Female community health volunteers
LMICs: Low- and middle-income countries
MAKS: Mental Health Knowledge Schedule
NGO: Non-governmental organisation
PPP: Purchasing power parity
RIBS: Reported and Intended Behaviour Scale
SDS: Social Distance Scale
ToT: Training of Trainers
